# Rationale Design for Anchoring Pendant Groups of Zwitterionic Polymeric Medical Coatings

**DOI:** 10.1021/acs.langmuir.4c01395

**Published:** 2024-06-12

**Authors:** Jia-Yin Chen, Kang-Ting Huang, Shuehlin Yau, Chun-Jen Huang

**Affiliations:** † Department of Chemical & Materials Engineering, National Central University, Jhong-Li, Taoyuan 320, Taiwan; ‡ R&D Center for Membrane Technology, Chung Yuan Christian University, 200 Chung Pei Rd., Chung-Li City 32023, Taiwan; § Department of Chemistry, National Central University, Jhong-Li, Taoyuan 320, Taiwan

## Abstract

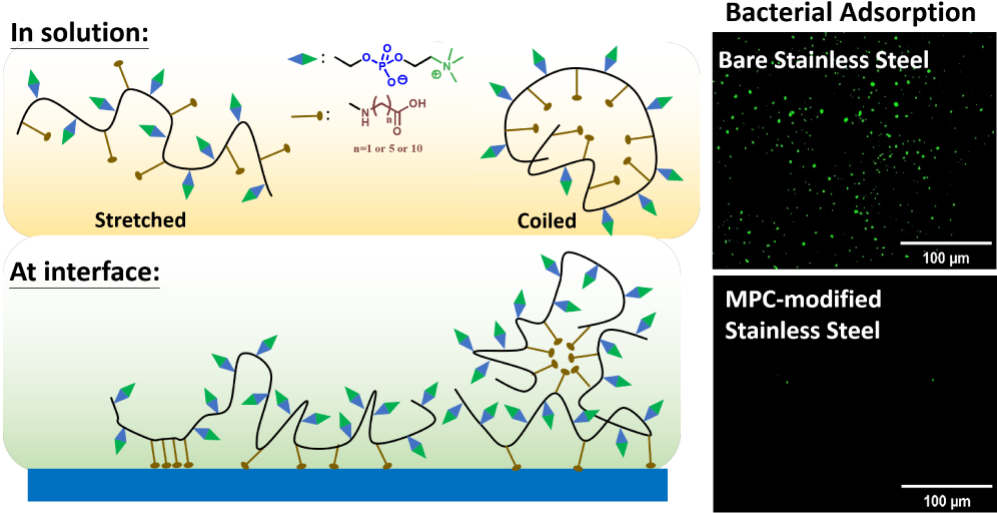

A biocompatible and antifouling polymeric medical coating was developed through rational design for anchoring pendant groups for the modification of stainless steel. Zwitterionic 2-methacryloyloxyethyl phosphorylcholine (MPC) was copolymerized individually with three anchoring monomers of carboxyl acrylamides with different alkyl spacers, including acryloylglycine (2-AE), 6-acrylamidohexanoic acid (6-AH), and 11-acrylamidoundecanoic acid (11-AU). The carboxylic acid groups are responsible for the stable grafting of copolymers onto stainless steel via a coordinative interaction with metal oxides. Due to hydrophobic interaction and hydrogen bonding, the anchoring monomers enable the formation of self-assembling structures in solution and at a metallic interface, which can play an important role in the thin film formation and functionality of the coatings. Therefore, surface characterizations of anchoring monomers on stainless steel were conducted to analyze the packing density and strength of the intermolecular hydrogen bonds. The corresponding copolymers were synthesized, and their aggregate structures were assessed, showing micelle aggregation for copolymers with higher hydrophobic compositions. The synergistic effects of inter/intramolecular interactions and hydrophobicity of the anchoring monomers result in the diversity of the thickness, surface coverage, wettability, and friction of the polymeric coatings on stainless steel. More importantly, the antifouling properties of the coatings against bacteria and proteins were strongly correlated to thin film formation. Ultimately, the key lies in deciphering the molecular structure of the anchoring pendants in thin film formation and assessing the effectiveness of the coatings, which led to the development of medical coatings through the graft-onto approach.

## Introduction

Metals are extensively used as materials for biomedical implants and surgical devices due to their unique properties of high corrosion resistance, biocompatibility, high wear resistance, and excellent mechanical properties. Therefore, implementations of metals are found in orthopedic reconstructions, fracture fixation, oral and maxillofacial reconstructions, and cardiovascular interventions.^1^ Stainless steel is an iron-based alloy that contains at least 12% chromium. The corrosion resistance of stainless steel is attributed to the formation of stable chromium oxide (Cr_2_O_3_), which is key to the biocompatibility of the steel. However, desirable surface properties of stainless steel are needed to adapt to the biological environment by increasing its antifouling properties, preventing biofilm formation, and imparting functionality for eluting a specific drug.^2^ Various physicochemical approaches are used to modify stainless steel. For chemical modification, an ad-layer requires functional groups, such as silanols, amines, carboxyls, or quinones, to react with oxide groups on the metal surfaces.^3^ A completely functional and stable surface modification relies on not only the coating chemistry but also the deposition strategy, which considers the solvophobicity,^4^ material conformation,^5^ exhaust rate,^6^ and incubation temperature and time.^7,8^

**Scheme 1:**
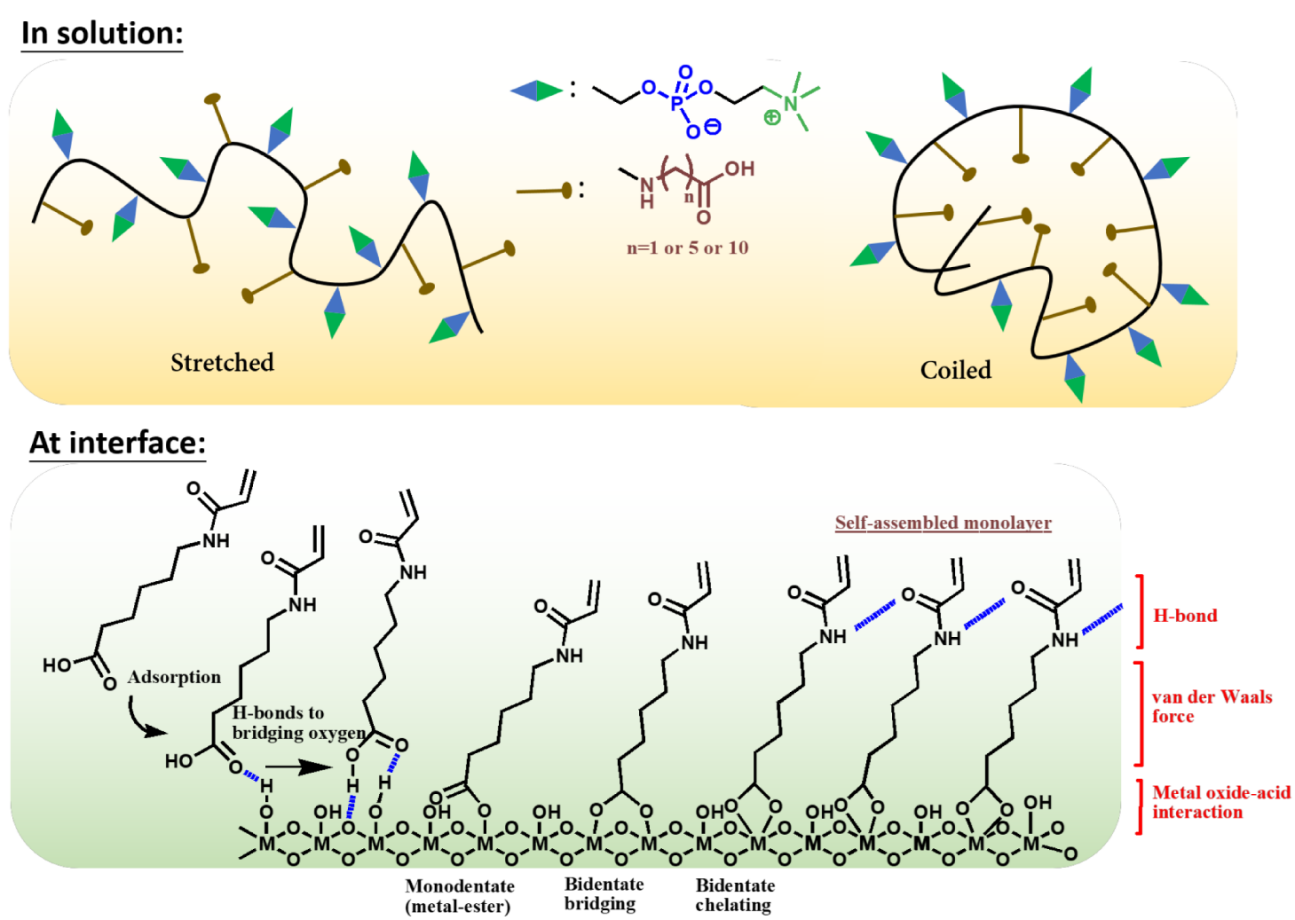
Schematic Illustration of Assembly Behaviors of MPC-based Copolymers in Solution and Adsorption of Acrylamide Fatty Acids at the Interface

Long-chain aliphatic carboxylic acids can adsorb on oxides to form organized and closely packed monolayer films, which are well-studied systems of self-assembled monolayers (SAMs).^9^ Because of wide availability and environmental friendly nature of carboxylates, carboxylic acid–based SAMs have been applied for various applications.^10,11^ Carboxylic acid groups are capable of binding to the metal oxide surface through”outer-sphere adsorption complexes” of hydrogen bonds and “inner-sphere adsorption complexes” of complex coordinative bonds (Scheme 1).^12,13^ The stable metal–oxide monodentate or bidentate monolayers are obtained upon annealing/curing by dehydration from the hydrogen bonds.^13−15^ In this work, 2-methacryloloxyethyl phosphorylcholine (MPC) was applied for the development of biocompatible and antifouling coatings. MPC is a bioinspired material with an identical polar group as the phospholipids in the cell membrane, enabling high wettability, lubrication, biocompatibility, and fouling resistance.^16−19^ Various poly(MPC)-based polymeric modifiers with different architectures were developed by copolymerization of MPC with alkyl vinyl compounds using conventional living radical polymerization techniques.^20−22^ The amphiphilic copolymers allow deposition on hydrophobic surfaces via physical adsorption to afford good anticoagulant and antifouling coatings.^22^ However, for long-term stability of the coatings, organic reactive components were introduced to copolymerization with MPC, allowing chemically grafting the polymeric coatings onto the organic or inorganic substrates.^23−27^ Moreover, due to the high polarity of the phosphorylcholine group, MPC-based amphiphilic copolymers tend to form aggregates depending on the solvent, molecular ratio, polymer concentration, and types of hydrophobic segments.^28,29^ The aggregation can lead to serious effects on the deposition behaviors, coverage, and functionality of the copolymers on substrates, which is generally overlooked.

Well-defined amphiphilic copolymers were developed by incorporating a monomer with a pendant fatty acid to investigate their pH-responsive property,^30−32^ formation of a polyion complex,^33^ and applications as a virus inhibitor^34^ and gelling agent.^31^ Copolymers composed of anionic/zwitterionic monomers and 11-acrylamidoundecanoic acid (11-AU) were found to modulate the formation and collapse of the unimer micelles depending on pH, polymer architecture, and composition.^30−32^ The pendant carboxylate group in the 11-AU unit becomes hydrophobic through selective protonation, leading to the formation of unimer micelles due to intramolecular hydrophobic interactions under acidic conditions in water. Herein, we aimed to develop antifouling and biocompatible medical coatings on stainless steel by using MPC-based copolymers. The anchoring moiety of the copolymers is carboxylic acid, responsible for stably grafting via coordinative interaction between carboxylic acid and metal oxides. The copolymers were synthesized with acrylamide aliphatic acids with various carbon lengths (Scheme 1) to systematically investigate the effects of molecular assembling behaviors at a solid substrate and in a coating solution on the thin film formation, lubrication, and antifouling properties. In addition, the role of the hydrophobic domain in the acrylamide aliphatic acids on wettability was discussed. Accordingly, the work provides molecular insight into the development of polymeric coatings via the graft-onto approach and explores rational design for the coating condition and formulation.

## Materials and Methods

### Materials

2-Methacryloyloxyethyl phosphorylcholine (MPC), 11-aminoundecanoic acid, and glycine were purchased from Sigma-Aldrich (St. Louis, MO, USA). 6-Acrylamidohexanoic acid was obtained from TCI, Inc. (Tokyo, Japan). Acryloyl chloride was purchased from Alfa Aesar (Ward Hill, Massachusetts, USA). 2,2′-Azobis(2-methylpropionitrile) (AIBN) was acquired from ECHO (Lake Zurich, IL, USA). Ammonium persulfate (APS) was purchased from Affymetrix (Santa Clara, California, USA). Luria–Bertani (LB) agar was obtained from AcuMedia (Lansing, Michigan, USA). Luria–Bertani (LB) broth was purchased from BD Bacto (Franklin Lakes, New Jersey, USA). The LIVE/DEAD BacLight Bacterial Viability Kit was purchased from Life Technologies (Carlsbad, California, USA). Bovine serum albumin (BSA) was purchased from Acros Organics. Anti-albumin antibody and goat anti-rabbit IgG antibody (HRP) were obtained from Arigo (Hsinchu, Taiwan). All of the other chemicals were reagent grade and used as received without further purification.

### Synthesis of Anchoring Monomers

#### Acryloylglycine (2-AE)

**Scheme 2:**
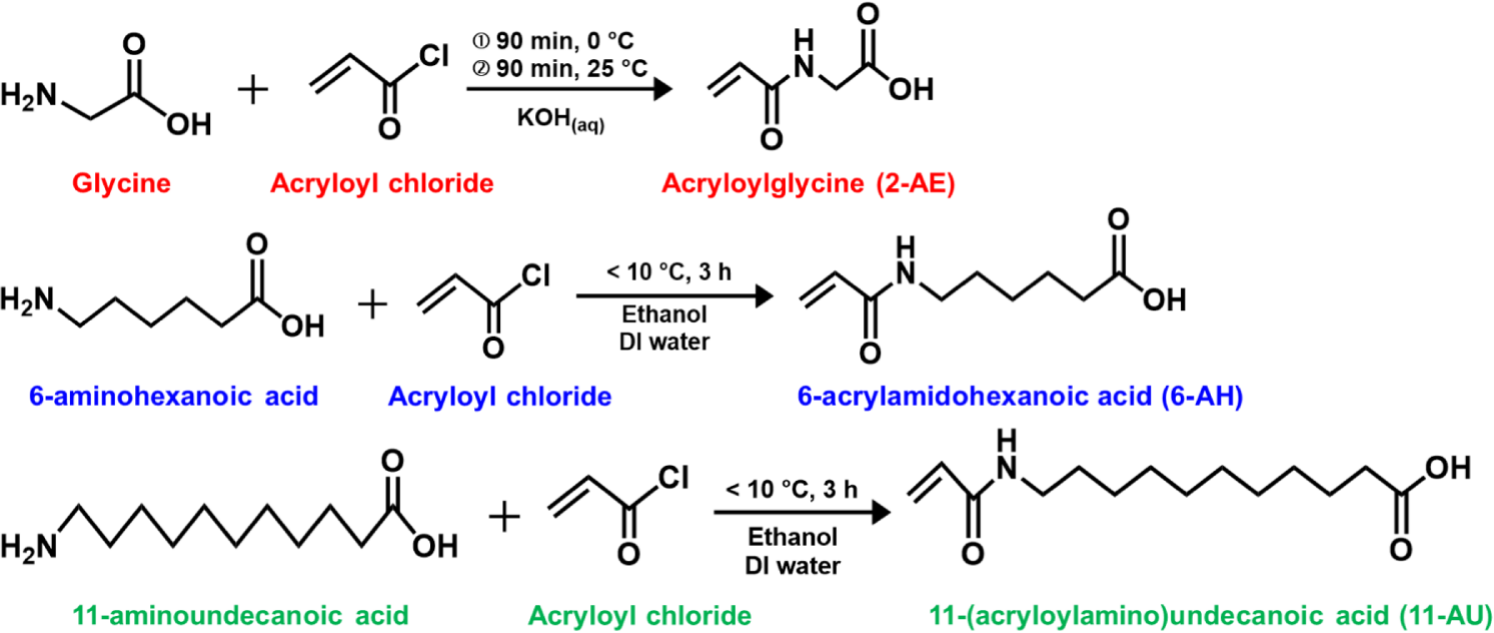
Synthesis of 2-AE, 6-AH, and 11-AU

Synthesis of 2-AE was referred to previous works.^35,36^ 2.2521 g of glycine (30 mmol) was dissolved in 30 mL of potassium hydroxide (1 M). The mixture was cooled at 0 °C with an ice water bath for 10 min. 2.9118 mL of acryloyl chloride (36 mmol) was added dropwise to the mixture using a dropping funnel. Afterward, the mixture is stirred for 90 min at 0 °C and then for 90 min at room temperature. The solution was then washed with diethyl ether (two times with 20 mL), and the aqueous phase is acidified to pH 2. The product was extracted with ethyl acetate (three times in 20 mL). The residue solvent is concentrated with a rotary evaporator to get an oily product. The synthesis processes for all anchoring monomers are shown in Scheme 2.

#### 6-Acryloylhexanoic Acid (6-AH)

An aqueous solution of ethanol (50 mL absolute ethanol and 5 mL distilled water) was used to dissolve 6-aminohexanoic acid (1.3118 g, 0.01 mol) and NaOH (1.2 g, 0.03 mol) with stirring. Acryloyl chloride (1.165 mL, 0.0144 mol) was added dropwise to the flask. After 3 h of stirring, the reaction mixture was acidified with HCl to pH 3. The acidified filtrate was then subjected to precipitation in acetone, resulting in the formation of a product that appeared as a white powder.^37^

#### 11-(Acryloylamino)undecanoic Acid (11-AU)

Similar to the synthesis of 6-AH, an aqueous solution of ethanol (250 mL of absolute ethanol and 25 mL of distilled water) was used to dissolve 11-aminoundecanoic acid (10 g, 0.7623 mol). NaOH (6 g, 0.15 mol) was added slowly until a clear solution is obtained. Next, acryloyl chloride (6 mL, 0.0743 mol) was added dropwise and the reaction mixture was stirred for 3 h at 100 °C. After filtration, the filtrate was acidified with diluted hydrochloric acid and washed with triply deionized water. The white precipitate formed was collected after filtration.^38,39^

### Synthesis of Modifier Copolymers

According to previous work,^32^ copolymerization of MPC and 2-AE was carried out by conventional free-radical polymerization in the presence of thermo-initiator APS in deionized water. A 0.6 M monomer solution was prepared by weighing MPC, 2-AE, and APS in different molar ratios, as shown in Table 1. The deionized water solution was purged with *N*_2_(*g*) for 30 min. Copolymerization was carried out at 80 °C for 16 h.

**Table 1:**
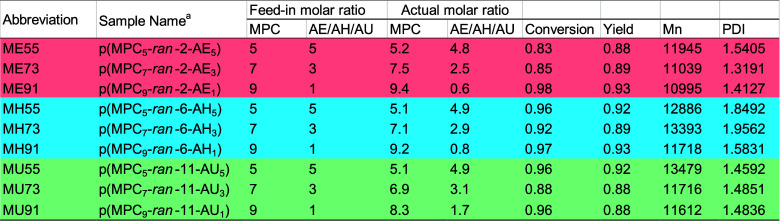
Molecular Ratios, Conversion, Yield, Mw, and PDI of Polymeric Modifiers^a^

a The suffix is the feed-in molar ratio of monomers.

Copolymerization of 2-methacryloyloxyethylphosphorylcholine (MPC) and 6-AH was conducted by free-radical polymerization in the presence of AIBN in methanol. A 0.4 M monomer solution was prepared by weighing MPC, 6-AH, and AIBN in different molar ratios, as shown in Table 1. The methanol solution was degassed in a vacuum line by three freeze–pump–thaw cycles. Copolymerization was carried out in N_2_ at 60 °C for 16 h.

Copolymerization of 2-methacryloyloxyethylphosphorylcholine (MPC) and 11-AU was carried out by ordinary free-radical polymerization in the presence of AIBN in methanol. A 0.6 M monomer solution was prepared by weighing 2-methacryloyloxyethylphosphorylcholine (MPC), 11-AU, and 2,2′-azobis(2-methylpropionitrile) (AIBN) in different molar ratios, as shown in Table 1. The methanol solution was outgassed on a vacuum line by three freeze–pump–thaw cycles. Copolymerization was carried out at 60 °C for 16 h.^33^

**Scheme 3:**
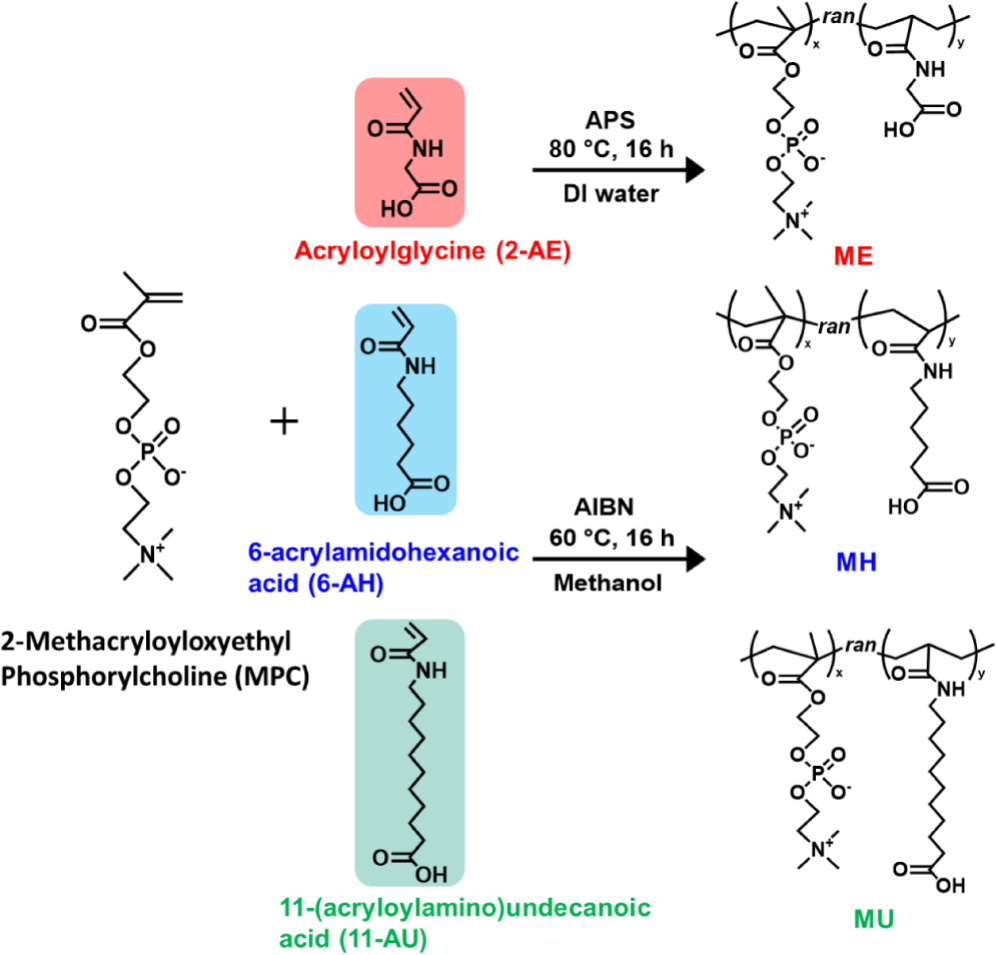
Synthesis of Copolymers of ME, MH, and MU

All copolymers were purified by dialysis against ethanol and freeze-drying to obtain white powders for further analysis and surface modification. The synthesis procedures were depicted in Scheme 3.

### Surface Modification of Stainless Steel

In addition to 2-AE and ME, the other molecules and copolymers exhibit good solubility in methanol but are insoluble in deionized water. Thus, to avoid formation of large copolymer aggregates, ME series were dissolved in deionized water and coating solutions for MH and MU series were prepared in methanol. The modification was realized by a solvothermal reaction in a Teflon-lined stainless steel autoclave.^40,41^ Generally, a clean stainless steel-grade 316 substrate was immersed in an autoclavable 30 mL bottle, which contained a modification solution with 1 wt % of copolymers (2-AE and ME in deionized water and the others in methanol). Then, the autoclave was heated to 100 °C for 2 h. After the autoclave was gradually cooled to room temperature, the modified SS316 was washed with methanol or deionized water and dried in a stream of nitrogen.

### Surface Characterizations

#### XPS

X-ray photoelectron spectroscopy (XPS) results were acquired using a Physical Electronics system with monochromatized Al Kα radiation (1486.6 eV). The C 1s spectrum was calibrated using the characteristic peak at 284.8 eV, followed by peak analysis using XPSPEAK 41. The Shirley method was employed to execute the baseline correction. Subsequently, data visualization was performed by using OriginPro 2021, accompanied by intensity normalization. The calculation of area under the curve was carried out using the “Mathematics Integrate” function within the Analysis tool.

#### Surface Wettability

The sessile drop method was used to statistically measure the in-air contact angles of the modified surfaces. A microsyringe was used to fix the volume of water droplets at 5 μL, and each sample was randomly recorded at at least three locations by an optical contact angle goniometer (Phoenix mini, Surface Electro Optics, Seoul, Korea).

#### Friction Test

The custom-designed friction testing apparatus was affixed to a universal testing machine. On the testing setup, a medical-grade silicone sheet with a hardness of 60 A was laid at the bottom of a water tank. Deionized water was introduced into the tank to completely cover the silicone sheet. In preparation for the testing, the modified stainless steel specimens were immersed in deionized water for 1 min. Subsequently, the stainless steel specimens were securely attached beneath the slider by using a robust double-sided adhesive. The testing involved subjecting the modified stainless steel specimens to underwater friction assessment under the conditions of a pulling speed of 150 mm/min and a displacement of 130 mm, encompassing a range of displacement percentages from 40% to 75%. The coefficient of dynamic friction (μ_k_) was then computed using the formula μ_k_ = *F* (sliding friction force)/*F*_N_ (normal force), wherein *F* represents the sliding friction force and *F*_N_ signifies the normal force.

#### Optical Film Thickness

The thicknesses of all coatings and polymer films on polished stainless steel were measured using an ellipsometer (alpha–SE, J.A. Woollam Co., US). The He–Ne laser with a wavelength of 632.8 nm was applied with three different incident angles of 65°, 70°, and 75°. At least three random spots on each substrate were measured before and after surface modification.

#### Cyclic Voltammetry (CV)

CV measures the electrochemical activities of a working electrode material toward the electrolyte. It gives information related to the mechanism during the electrodeposition process of the thin film including an electrochemical reaction and a precipitation reaction. In order to understand the electrodeposition process, the CV of the stainless steel substrate in an electrodeposition bath was performed over the potential window from 0.499 V to −0.8 V at a scan rate of 50 mV s^–1^. Utilizing a Ag/AgCl electrode as the reference electrode, in a 0.1 M PBS electrolyte solution, the obtained voltammogram was integrated to calculate the specific capacitance. Specific capacitance (*C*_p_) is often defined as the integration across the whole set of statistics per unit area of both the cathode and the anode. *C*_p_ is measured in *F*/*g*. To determine *C*_p_, the following equation was used. , where *A* = CV area, *m* = load, *k* = scan speed, and Δ*V* is the whole voltage range of the CV analysis. In this study, the *m*, *v*, and Δ*V* values were 10 mg, 0.05 V/s, and 1.299 V, respectively.

#### FTIR

The Fourier-Transform Infrared Spectrometer (Bruker, Vertex 80v, Germany) was employed to record FTIR spectra of modified substrates with a resolution of 1 cm^–1^. Experiments were performed under an infrared beam with an incidence angle of 80° using PIKE Technologies 80Spec (USA) to obtain the nanoscale-analyzed surface.

### Small Angle X-Ray Scattering (SAXS)

To investigate the aggregation behavior of the copolymers in water and methanol, SAXS measurements for 1 mg/mL aqueous solutions were carried out. To evaluate the size of the aggregates, we fitted the experimental profiles with theoretical curves. The majority of the patterns were collected over a scattering vector range of 0.01–0.4 Å^–1^. The length of the scattering vector, *q*, is given by *q* = (4π/λ) sin θ, where θ is half the scattering angle. For calculations, a spherical polymer–micelle model was assumed as the micelle structure. The model was described in detail in previous literatures.^42,43^

### Fouling Test with Bacteria

In separate 50 mL centrifuge tubes, 10 μL of *Staphylococcus aureus* (*S. aureus*, ATCC 14 990, Gram-positive) and *Escherichia coli* (*E. coli*, ATCC 25 922, Gram-negative) were individually added to 30 mL of LB broth culture medium. The tubes were then placed in 37 °C, shaken at 110 rpm, and cultured for 16 h. Subsequently, the bacterial cultures were centrifuged at 5000 rpm for 5 min; the supernatant was removed, and the pellet was resuspended in sterile PBS. The bacterial cultures were then diluted to an optical density (OD600) of 0.1. The modified stainless steel samples to be tested were placed in a 12-well plate and were incubated at 37 °C for 3 h. The nonadherent bacteria were washed at 37 °C and 120 rpm for 5 min with sterile PBS, with this step repeated three times. Following staining with LIVE BacLight at room temperature for 15 min, excess stain was removed by washing with sterile PBS at 37 °C and 120 rpm for 5 min. The surplus dye was thoroughly removed. Finally, the stained modified substrates were observed by using a fluorescence microscope. At least three random positions were captured for each sample. ImageJ software was employed to quantify and analyze the bacterial colonies.

For scanning electron microscope (SEM) imaging, substrates were washed three times with sterilized PBS solution after immersion in the bacteria solution. Then, it is fixed with 2.5% glutaraldehyde solution for 12 h at 4 °C. After that, the samples were dehydrated gradually with a series of ethanol–water solutions (25 vol%, 50 vol%, 75 vol%, 95 vol%, and 100 vol%, each for 20 min). Then, the samples were dried under vacuum conditions and observed by SEM (HITACHI, S-800).

### Fouling Test with Protein

Unmodified and modified stainless steel samples were placed within a 12-well plate, with each well receiving 1 mL of PBS. The plate was incubated at a temperature of 37 °C and a shaking rate of 80 rpm for 30 min. The agitation allowed the expansion of MPC polymer chains on the stainless steel surface, optimizing their resistance to fouling. A solution of BSA was prepared in PBS at a concentration of 4.5 mg/mL. The PBS in the wells was replaced with 1 mL of the protein solution, and cocultivation was carried out at 37 °C and 80 rpm for 3 h. A PBST solution was prepared by combining PBS with 0.05% Tween 80 in a volume ratio. After cocultivation, the protein solution was removed, and 1 mL of PBST was added to the wells. The nonadherent protein was washed at 37 °C and 80 rpm for 5 min, with this process repeated three times. A primary antibody solution of anti-BSA IgG was prepared in PBST at a concentration of 5.5 μg/mL. Each well received 1 mL of the secondary antibody solution, and cocultivation was performed under conditions of 37 °C and 80 rpm for 1 h. During this phase, binding between anti-BSA IgG and BSA adsorbed on the stainless steel surface occurred. After cocultivation, the solution containing the primary antibody was replaced with 1 mL of PBST, and a 5 min wash was repeated three times at 37 °C and 80 rpm.

A secondary antibody solution of HRP was prepared in PBST at a concentration of 5.5 μg/mL. The PBST was replaced with 1 mL of the secondary antibody solution, and cocultivation was performed at 37 °C and 80 rpm for 1 h. At this stage, HRP secondary antibodies were bound with anti-BSA IgG antibodies. Following cocultivation, the solution containing the secondary antibody was replaced with 1 mL of PBST, and a 5 min wash was repeated three times at 37 °C and 80 rpm. The enzyme–substrate complex 3,3′,5,5′-tetramethylbenzidine (TMB) was utilized as the chromogenic agent for HRP. Under HRP catalysis, TMB produced a soluble blue product. Upon completing the wash steps, samples were transferred to a clean 12-well plate. Each well was provided with 1 mL of TMB. After a 3 min reaction, 1 mL of 1 M sulfuric acid solution was added to each well to terminate the TMB color reaction. Finally, the solutions were transferred to a 96-well plate, and the OD values of the samples were measured at a wavelength of 450 nm using an enzyme immunoassay analyzer.

### Statistical Analysis

The data were reported as the means ± standard deviation (SD) or standard error of the mean (SEM). Student’s *t* test was utilized to determine the statistical analyses among different groups. The probabilities of *p* ≤ 0.05 were considered as a significant difference. Origin 9.0 software (OriginLab Corporation, MA, USA) was employed for statistically analyzing all experimental data.

## Results and Discussion

### Synthesis and Characterization of Anchoring Monomers

Three anchoring acrylamide monomers with different alkyl spacers, i.e., 2-AE, 6-AH, and 11-AU, were synthesized by the reaction of acyl chloride with amines as described in the Materials and Methods section. Products of 2-AE, 6-AH, and 11-AU were harvested and purified. ^1^H NMR spectroscopy was performed to confirm the chemical structures of the monomers. The spectra of monomers are presented in Figure S1. Assignments for the ^1^H NMR chemical shifts of the monomers are presented: for 2-AE (600 MHz, DMSO-d, δ, ppm): 6.33–6.25 (CH_2_CH, 1H), 6.14–6.07 (CH_2_CH, 1H), 5.64–5.6 (CH_2_CH, 1H), 3.85–3.81 (NH_2_CH_2_COOH, 2H); for 6-AH (600 MHz, methanol-d, δ, ppm): 6.26–6.17 (CH_2_CH, 2H), 5.65–5.62 (CH_2_CH, 1H), 3.27–3.23 (NH_2_CH_2_(CH_2_)_3_CH_2_COOH, 2H), 2.32–2.28 (CH_2_COOH, 2H), 1.67–1.34 (NH_2_CH_2_(CH_2_)_3_CH_2_COOH, 6H); for 11-AU (600 MHz, methanol-d, δ, ppm): 6.26–6.16 (CH_2_CH, 2H), 5.65–5.62 (CH_2_CH, 1H), 3.26–3.21 (NH_2_CH_2_CH_2_CH_2_CH_2_, 2H), 2.29–2.25 (CH_2_COOH, 2H), 1.67–1.34 (NH_2_CH_2_(CH_2_)_8_CH_2_COOH, 16H).

**Figure 1:**
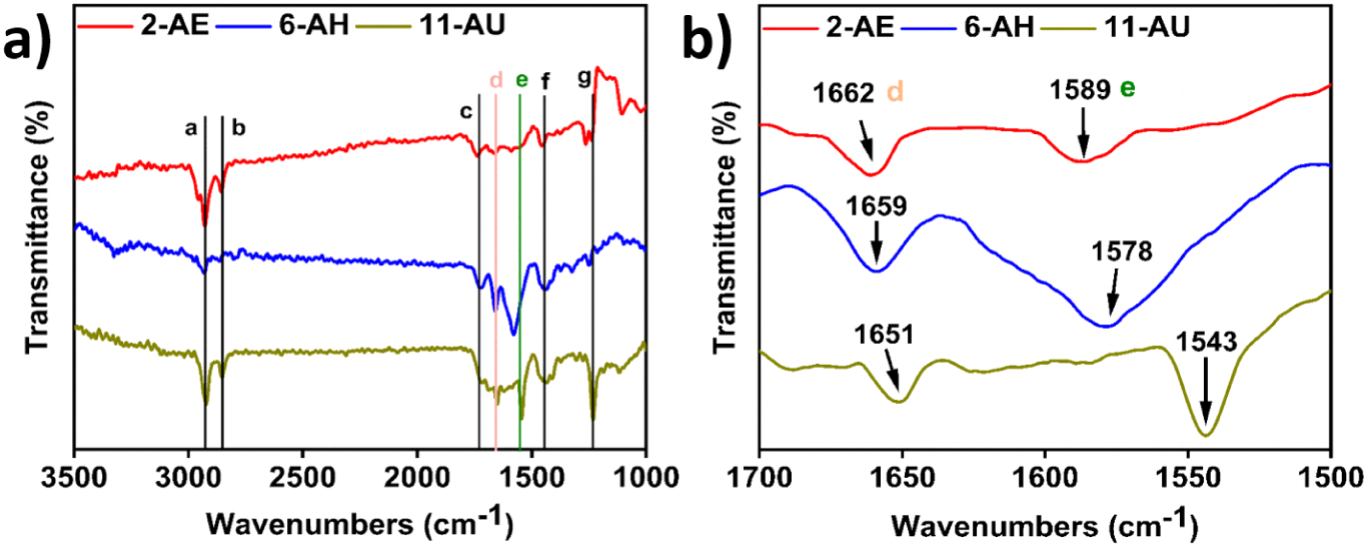
FTIR equipped with a grazing angle accessory was applied for analysis of the stainless steel samples with the modification of 2-AE, 6-AH, and 11-AU by the solvothermal strategy (a). FTIR spectra in the region of the amide I (ν_(C=O)_) and amide II (δ_NH_+ ν_(C–N)_) modes (b).

To investigate the assembly behaviors of anchoring monomers on stainless steel substrates, 2-AE, 6-AH, and 11-AU were deposited on surfaces through chemical binding of carboxylic acids onto metal oxides by solvothermal strategy in an autoclave at 100 °C.^40^ The reaction processes in the solvothermal strategy include the formation of double hydrogen bonds between the carboxylic acid molecule and metal oxides, and the coordination at solvothermal temperature by dehydration from the hydrogen bonds.^40,44^ Because of its solubility, 2-AE was dissolved in deionized water, and 6-AH and 11-AU were prepared in methanol for surface modification. The monomer coatings on stainless steel were examined using FTIR equipped with a specular reflection accessory for grazing angle analysis in Figure 1.^45^ The FTIR spectra for bare stainless steel and monomer-modified samples are presented in Figure 1a, and assignments for the characteristic transmittance peaks are listed in Table S1. The presence of carboxylic acid and amide groups for all anchoring monomers on stainless steel surfaces clearly demonstrates their adsorption to form stable thin films using the solvothermal strategy.

We further analyzed the FTIR spectra in the region of amide I (ν_C=O_) and amide II (δ_NH_ + ν_C–N_) modes for anchoring monomer-treated stainless steel samples to understand the intermolecular interactions of monomers in Figure 1b. The anchoring monomers of 2-AE, 6-AH, and 11-AU contain internal amide groups. The frequencies of the amide I (ν_C=O_) vibration, amide II (δ_NH_ + ν_C–N_) vibration, and the N–H stretch vibration are known to depend upon the hydrogen bonding state.^46,47^ The wavenumber difference (Δν) between amide I (ν_C=O_) and amide II (δ_NH_ + ν_C–N_) modes is correlated with the strength of the intermolecular hydrogen bonds.^48^ The smaller the Δν, the stronger the hydrogen bonds between adjacent amide groups.^49,50^ From the FTIR measurements in Figure 1b, the order of the Δν of the coatings is 2-AE (Δν = 73 cm^–1^) < 6-AH (Δν = 81 cm^–1^) < 11-AU (Δν = 108 cm^–1^). Therefore, the anchoring monomer of 2-AE deposited on stainless steel forms the strongest hydrogen bonding compared with the other monomers. The hydrogen bonds between amide groups can serve as an important driving force for the ordered alignment of 2-AE on surfaces.

**Figure 2:**
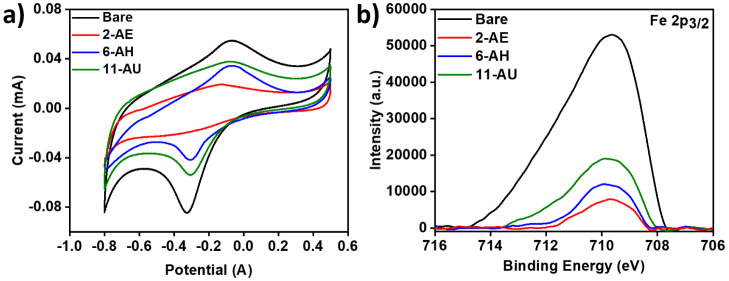
Cyclic voltammetry analysis of stainless steel samples with and without modification of 2-AE, 6-AH, and 11-AU (a), recorded at 50 mV/s in 0.1 M PBS. XPS spectra showing Fe 2p_3/2_ emission from stainless steel samples with and without these modifiers (b).

Cyclic voltammetry (CV) and X-ray photoelectron spectroscopy (XPS) were used to analyze the thin film formation of anchoring monomers on stainless steel. Figure 2a shows cyclic voltammograms obtained on a bare stainless steel substrate and the corresponding curves for modified substrates. Measures of the peak area on cyclic voltammetric curves allowed for the comparison of the packing density of the thin films on metals. The areas for bare and 2-AE-, 6-AH-, and 11-AU-modified substrates were 0.0747, 0.0291, 0.0366, and 0.0574 mA·V (or C/cm^2^ if these values refer to the area under the *j*–*E* curves), respectively. These values were used to calculate the surface coverages of 61%, 51%, and 23% for 2-AE, 6-AH, and 11-AU on the stainless samples, respectively. These results imply that 2-AE film was the densest and could be closely packed on the sample, which impeded electron transfer and resulted in the lowest current.^48,51^ In Figure 2b, XPS spectra were collected from bare steel and samples modified with anchoring molecules. The Fe 2p_3/2_ binding energy intensity is inversely proportional to the coating thickness and density.^52^ The higher the signal intensity, the thinner and denser the coating layer. These voltammetric and XPS results consistently show that the 2-AE film was the densest among these and could best shield the substrate steel.

The major contributing factors for having a dense 2-AE on stainless steel are the coordinative binding of carboxylic acids onto metal oxides and internal hydrogen bond networks between amide groups.^53^ The van der Waals forces between alkyl spacers of 11-AU did not seem to help the formation of a tightly packed and well-ordered thin film on the steel. Factors such as a large radius of gyration and poor head–tail conformational reorganization and alignment were also important.^54^

### Synthesis and Characterizations of Bioinspired Zwitterionic Polymeric Modifiers

Bioinspired biocompatible monomer, MPC, was utilized to copolymerize with anchoring monomers to afford polymeric modifiers for surface modification of stainless steel. MPC is a zwitterionic material, containing two ionic groups with opposite charges to keep it electrically neutral and hydrated.^55,56^ MPC has been applied for the surface modification of various medical devices, such as artificial joints and contact lenses.^18,56^ However, due to its hydrophilicity, poly(MPC) cannot adhere to solid surfaces through physical adsorption. Therefore, in this work, we aim to develop biocompatible antifouling MPC polymeric modifiers via free radical polymerization to chemically attach them through coordinative interactions with metallic surfaces. Three anchoring monomers were included in the random copolymers with various feed-in molar ratios to explore the most effective antifouling coating for stainless steel. The chemical structures and compositions of copolymers were examined by NMR in Figure S1. As shown in Table 1, the notation, molecular ratios, conversion, yield, molecular weight (Mw), and polydispersity index (PDI) for all copolymers were summarized.

**Figure 3:**
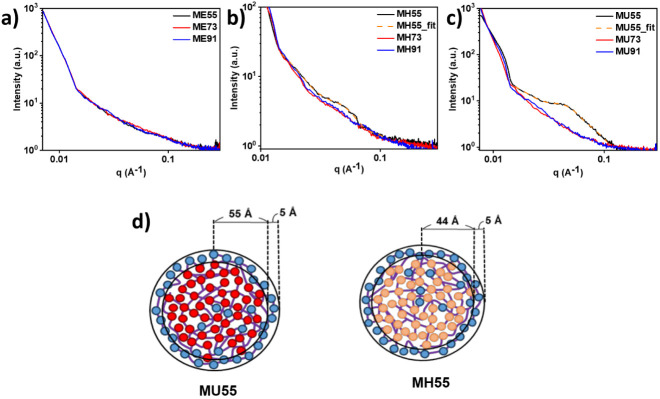
SAXS profiles (I(q)) of polymeric modifiers at a concentration of 1 wt %. ME series (a) were dissolved in deionized water, and coating solutions for the MH (b) and MU (c) series were prepared in methanol. (d) Schematic illustration for the core–shell spherical morphologies for MU55 and MH55.

The self-assembly behavior of these polymeric modifiers in solutions was investigated using SAXS. To avoid the formation of large copolymer aggregates, the ME series was dissolved in deionized water, and coating solutions for the MH and MU series were prepared in methanol. A 1 wt % concentration of copolymers was dissolved in corresponding solvents. We used coating solutions for SAXS measurements and thin film formation on steel. The scattering patterns recorded for all copolymers in solutions are shown in Figure 3a–c. We observed that the patterns for MH55 and MU55 show an upturn in scattering intensity at *q* < 0.1 Å^–1^. The second maximum in the scattering intensity, in addition to the first maximum at *q* < 0.01 Å^–1^ for forward scattering, is generally characteristic of core–shell structures.^57^ To evaluate the size of the aggregates, we fitted the experimental profiles by a theoretical model described in the literature.^42,43^ The spherical core–shell model was assumed, with a core–shell spherical morphology as shown in Figure 3d, where the central core consisted of 6-AH for MH55 or 11-AU for MU55 segments and the outer shell consisted of the MPC segment swollen by methanol. The radii of the hydrophobic cores of MU55 and MH55 micelles are approximately 55 and 44 Å, respectively, with both hydrophilic shell thicknesses of about 5 Å. Herein, the copolymers with a higher portion and chain length of alkyl groups enable spontaneous micelle aggregation. Micelle aggregation is ruled by two major contributions to the free energy. The embedded hydrophobic moieties in the hydrophobic core have a favorable entropic contribution to the free energy.^58,59^ This effect favors large aggregates and phase separation.

### Deposition and Surface Characterizations of Polymeric Modifiers

**Figure 4:**
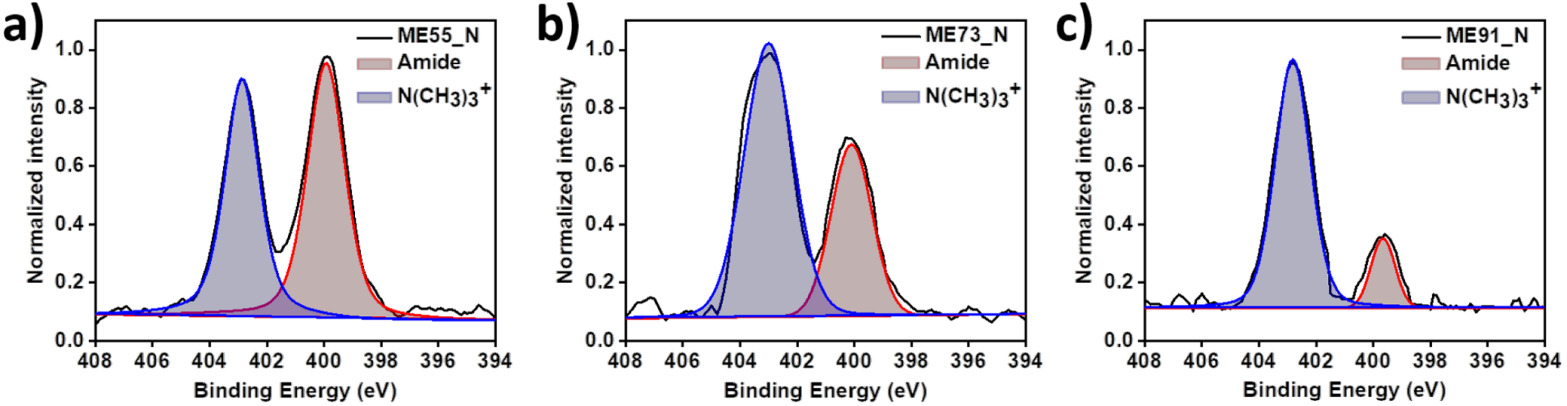
XPS measurements for the element N for the samples of ME55 (a), ME73 (b), and ME91 (c).

Polymeric modifiers were deposited on stainless steel surfaces by a solvothermal reaction. The steel samples were then cleaned to remove unattached polymers and dried in a stream of nitrogen. XPS measurements were conducted to determine the chemical compositions of the modified samples. The representative N 1s spectra for the ME series are shown in Figure 4a–c. There are two characteristic nitrogen species in the copolymers: MPC contains a quaternary ammonium (N(CH_3_)_3_^+^) group, while all anchoring monomers (2-AE, 6-AH, and 11-AU) contain amide (−C(=O)–N−) groups. Therefore, in the N 1s spectra, two distinct peaks are observed, centered at binding energies (BEs) of 403.0 and 400.2 eV for quaternary ammonium and amide, respectively. We calculated the ratio of integrated peak areas of these two characteristic peaks, which reveals the molar ratio of the corresponding monomers in the adhered modifiers. In Table 2, generally, the molar ratios from XPS spectra are approximately consistent with the feed-in molar ratios for the polymer synthesis. As a result, the chemical compositions of the polymeric coatings are confirmed for further analysis.

**Table 2:** Integrated Area Ratio between Quaternary Ammonium and Amide for Adhered Polymeric Modifiers

				polymeric modifiers					
	ME55	ME73	ME91	MH55	MH73	MH91	MU55	MU73	MU91
area ratio for MPC	0.533	0.671	0.863	0.546	0.649	0.862	0.475	0.657	0.872
area ratio for ADAH/AU	0.467	0.329	0.137	0.454	0.351	0.138	0.525	0.343	0.128

**Figure 5:**
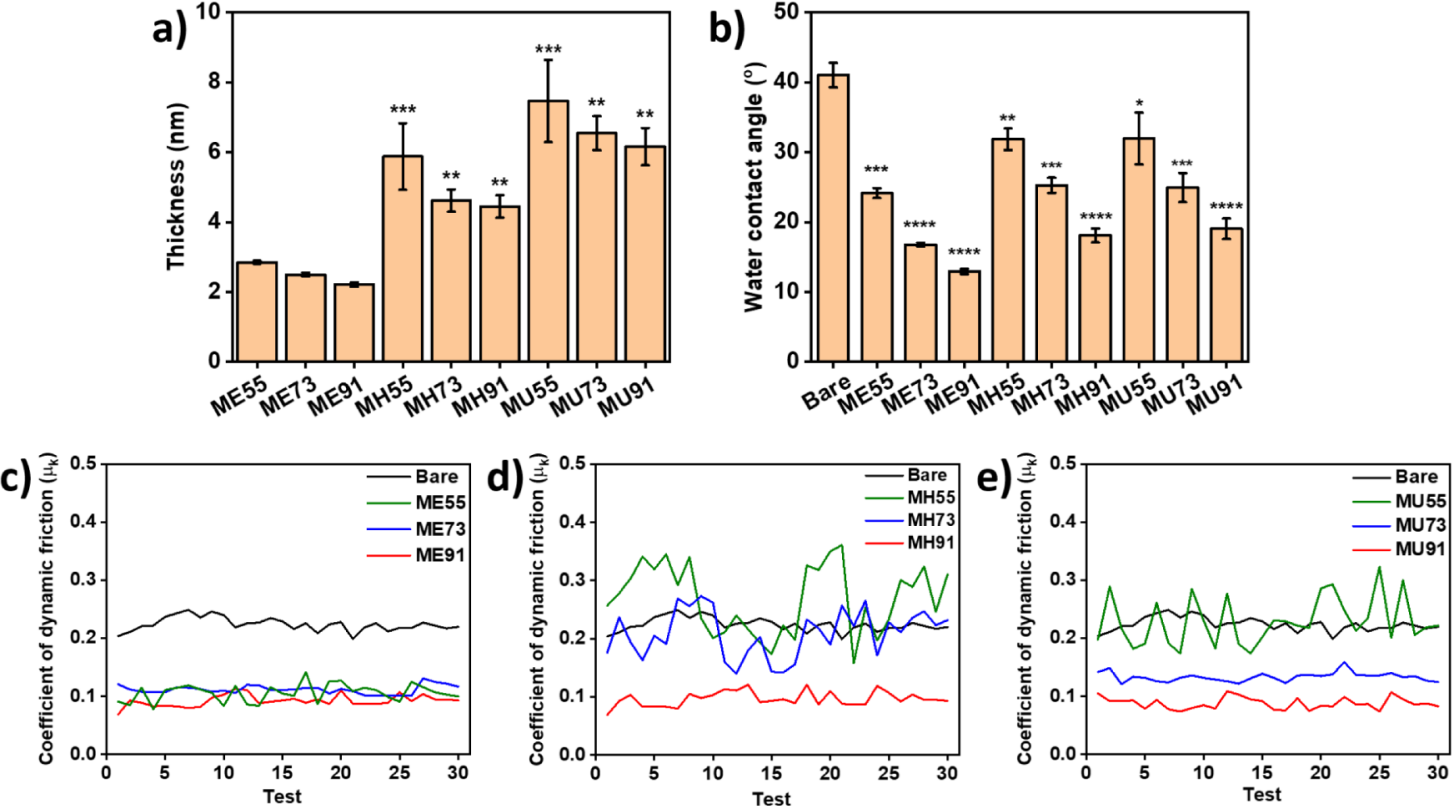
Surface characterizations for the polymeric coatings: ellipsometric thickness measurements (a), static water contact angle measurements (b), and surface friction tests (c–e). Statistical analysis: * *p* ≤ 0.05, ** *p* ≤ 0.01, *** *p* ≤ 0.001, and **** *p* ≤ 0.0001.

The modified samples were characterized by using ellipsometry, a contact angle goniometer, and a universal test machine for film thickness, wettability, and lubrication, respectively. In Figure 5a, the measurements of film thickness of modifiers on polished stainless steel show that the coatings of the ME series are thinner than others. Additionally, the thickness of the coatings slightly increases with the molar ratios of the anchoring monomers in the copolymers, which can be ascribed to the more aggregated structures in the films, as found in SAXS measurements. Because of the zwitterionic nature of the MPC, the modified samples should become hydrophilic. In Figure 5b, the water contact angles of the coatings are all below 35°, consistent with results in the literature.^16,60,61^ Interestingly, when the molar ratios of the anchoring monomers are higher, the wettability of the films becomes poorer, which can be rationalized by the presence of more hydrophobic alkyl chains and fewer zwitterionic groups.

Moreover, MPC has been applied to medical apparatus as a lubricant coating.^19,62^ Herein, a tribological analysis for the MPC-based modifiers on stainless steel samples was conducted. In the testing setup, the modified stainless steel samples were securely attached beneath the slider and immersed in deionized water. A medical-grade silicone sheet with a hardness of 60 A was laid at the bottom of a water tank to contact the modified surfaces. In Figure 5c–e, the bare sample exhibited a high coefficient of dynamic friction, indicating poor lubrication property. After modification with polymers with the highest MPC molecular ratios (i.e., ME91, MH91, and MU91) and those copolymerized with the 2-AE anchoring monomer (i.e., ME91, ME73, and ME55), the coefficients of dynamic friction reduce to around 0.1 μ_k_, even after repeated tests for 30 cycles. The results reveal the good slippery property of the MPC and good wear resistance of the coatings against the silicone rubber. However, the coatings with higher molecular ratios and larger alkyl spacer of anchoring monomers demonstrated higher friction, which should be attributed to the incomplete coverage of MPC and the interaction between the silicone and the exposed hydrophobic anchoring segments and/or steel substrate. Overall, ME polymeric modifiers provide thin and dense coatings on stainless steel for high wettability and robust lubrication, attributed to the well-stretched polymer chain permitting the full coverage and strong adhesion of ME polymers. On the other hand, particularly for MU55 and MH55, because of the hydrophobic aggregation and high exposure of alkyl structures, the deposition of the polymeric modifiers results in inadequate film formation on the steel surface.

### Antifouling Properties of Polymeric Modifiers

**Figure 6:**
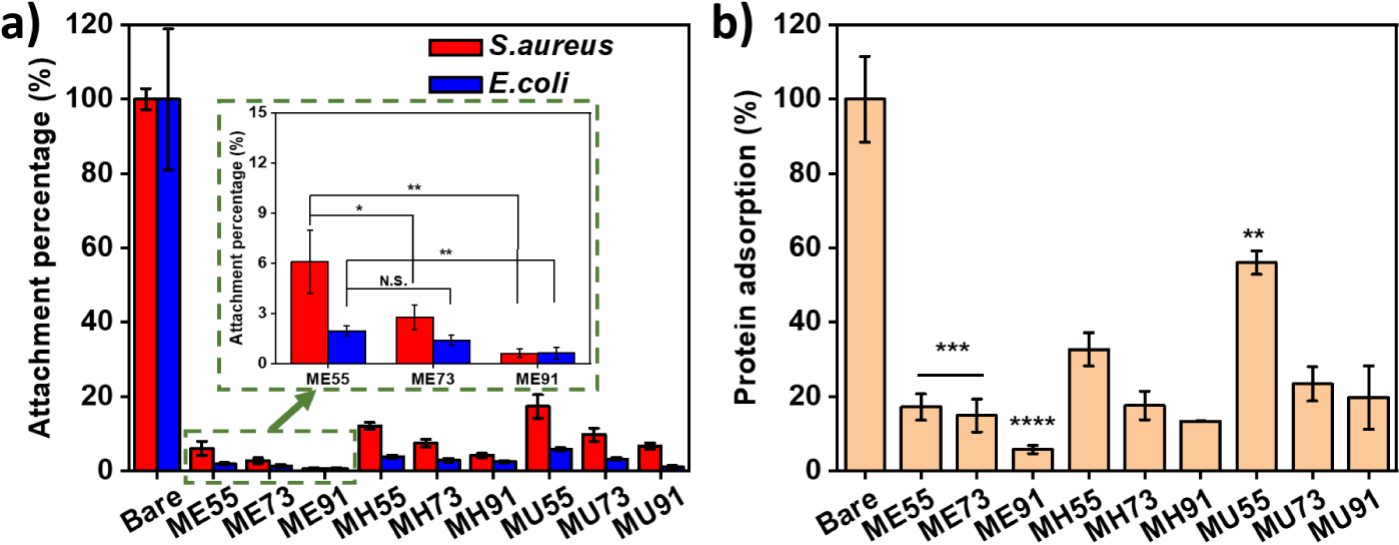
Biofouling tests on bare and modified stainless steel. (a) Bacterial adsorption of *S. aureus* and *E. coli* on samples. (b) BSA protein adsorption on samples. Statistical analysis: * *p* ≤ 0.05, ** *p* ≤ 0.01, *** *p* ≤ 0.001, and **** *p* ≤ 0.0001.

Because of its charge balance and hydrophilicity, MPC is regarded as an excellent biocompatible antifouling material for medical applications. In this work, foulants of bacteria (*S. aureus* and *E. coli*) and protein (BSA) were used to test the antifouling properties of polymeric modifiers on steel substrates. Biomaterial-associated infections (BAI) are one of the most common complications in medical practice,^63^ typically stemming from the attachment of microorganisms to the surfaces of medical devices. *S. aureus* and *E. coli* are commonly found in BAI.^64,65^ In the bacterial fouling tests (Figure 6a), the attachment percentage on the coatings was determined with respect to the bacterial number on the bare substrate. Representative fluorescence images for bacterial adsorption on bare and ME91-modified steel are shown in Figure S2. Basically, the percentage increases with the molar ratio of anchoring monomers in the polymers, which is likely due to the presence of cationic unattached carboxyl groups and hydrophobic spacers. Herein, ME91 demonstrated excellent fouling resistance against both strains of bacteria. In the protein fouling tests (Figure 6b), the protein adsorption (5.8% ± 1.1%) on ME91 was significantly lower than on other coatings. Moreover, long-term antifouling properties of coatings on stainless steel substrates against *E. coli* adsorption were accessed (Figure S3). After 1-week incubation of samples with bacteria, the adsorption of *E. coli* on ME91 was still lower than that of the other samples, which encourages implementation of medical coatings for implantable devices. The complete coverage by MPC and the stable adhesion of 2-AE in ME91 are major contributors to the outstanding antifouling properties of the polymeric modifier. The morphologies of *E. coli* on bare and ME91 in SEM images (Figure S4) reveal no obvious difference, indicating surface passivation by ME91.

## Conclusions

Biocompatible and antifouling polymeric medical coatings for stainless steel were developed by copolymerizing MPC and anchoring carboxyl acrylamides with different spacers. The copolymers were deposited on the surfaces by a solvothermal reaction through a coordinative interaction between carboxylic acid and metal oxides. The assembly behaviors of the anchoring monomers on steel substrates were analyzed, revealing robust hydrogen bonding within the internal amide groups of 2-AE. Moreover, copolymers such as MU55 and MH55, with anchoring moieties containing long carbon spacers, exhibited core–shell spherical morphologies in the coating solutions. Consequently, the substantial aggregation and hydrophobic domain of MU and MH copolymers contributed to an increased film thickness, elevated water contact angle, and reduced lubrication. ME91, owing to its high grafting density and hydrophilicity, displayed exceptional antifouling properties against bacterial and protein adsorption. Thus, this study underscores the influence of molecular assembly behaviors and polymeric structures on thin film formation and their respective functionalities.

## Supporting Information Available

^1^H NMR spectra of anchoring monomers (Figure S1); assignments of characteristic peaks of FTIR spectra (Table S1); representative fluorescence images for bacterial adsorption on bare and ME91-modified steel (Figure S2); long-term *E. coli* adsorption on bare and modified stainless steel samples; SEM images of *E. coli* on bare and ME91-modified stainless steel samples (Figure S4) (PDF)
