# Supplementary material for: Rationale Design for Anchoring Pendant Groups of Zwitterionic Polymeric Medical Coatings
Source: Langmuir. 2024 Jun 12;40(25):13236–46. doi: 10.1021/acs.langmuir.4c01395 (PMC11210289; doi:10.1021/acs.langmuir.4c01395)
Supplement: Supplementary file 1 — la4c01395_si_001.pdf [file la4c01395_si_001.pdf]

Supporting Information

Rationale Design for Anchoring Pendant Groups of Zwitterionic Polymeric Medical Coatings

Jia-Yin Chen, [a,#] *Kang-Ting Huang,* [a,#] *Shuehlin Yau,* [c*] *Chun-Jen Huang* [a, b *]

[a] Department of Chemical & Materials Engineering, National Central University, Jhong-Li, Taoyuan 320, Taiwan.

[b] R&D Center for Membrane Technology, Chung Yuan Christian University, 200 Chung Pei Rd., Chung-Li City 32023, Taiwan.

[c] Department of Chemistry, National Central University, Jhong-Li, Taoyuan 320, Taiwan.

[#]Equal contribution to the work.

* Corresponding author. E-mail: yau6017@ncu.edu.tw (SY); cjhuang@ncu.edu.tw (CJH)

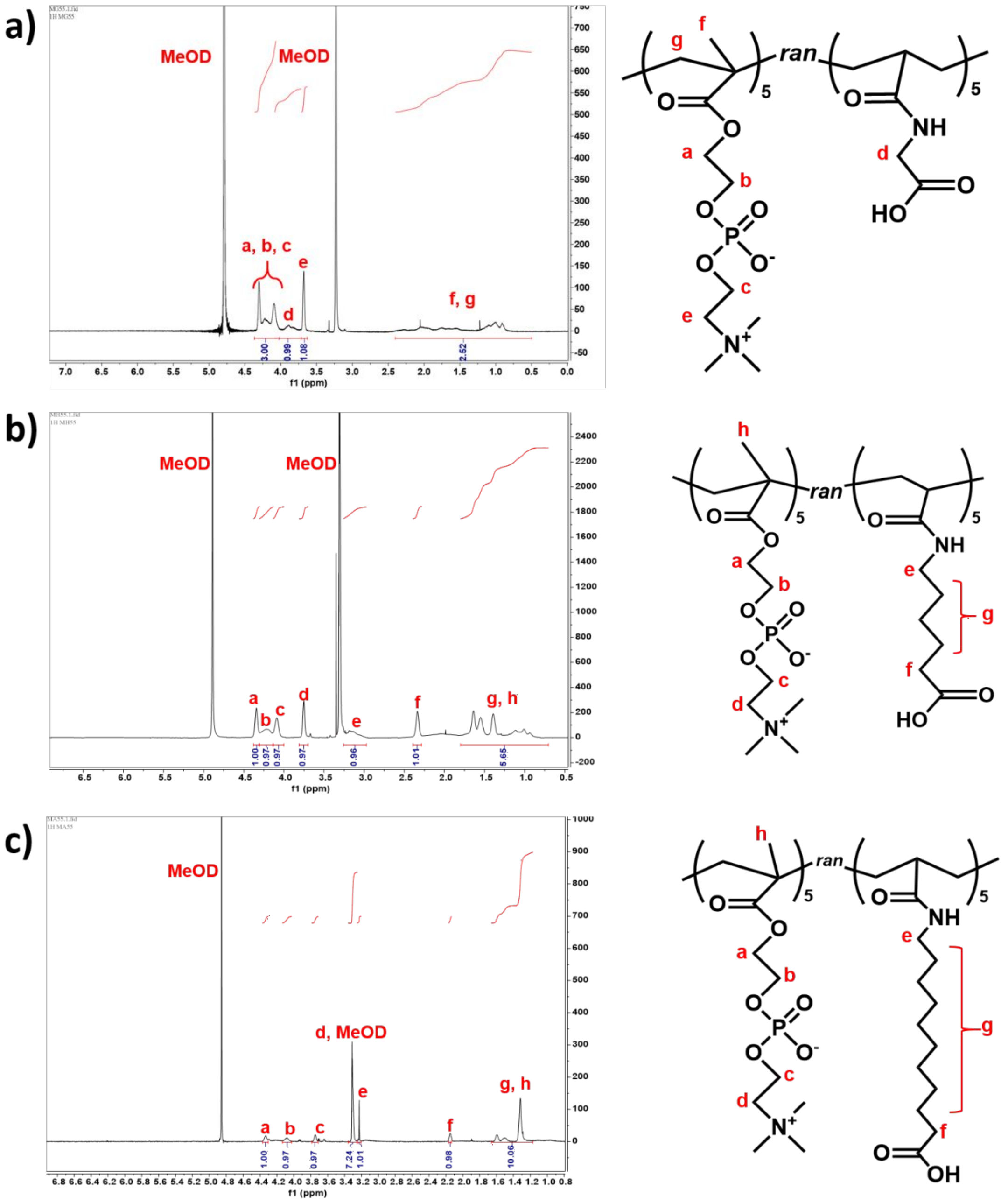

Figure S1. The ^{1}H-NMR spectra for (a) 2-AE in DMSO-d_6 and (b) 6-AH and (c) 11-AU in MeOD.

Table S1. Assignments of Characteristic Peaks of FTIR Spectra.

Peak symbol	Wavenumbers (cm^{-1})	Assignment
a	2926	CH_2 (Alkyl chain)
b	2854	
c	1735	C=O (Carboxylic acid)
f	1458	C-O (Carboxylic acid)
d	1651, 11-AU	C=O (Amide)
	1659, 6-AH	
	1662, 2-AE	
e	1543, 11-AU	C-N & N-H (Amide)
	1578, 6-AH	
	1587, 2-AE	
g	1230	C-C (Alkyl chain)

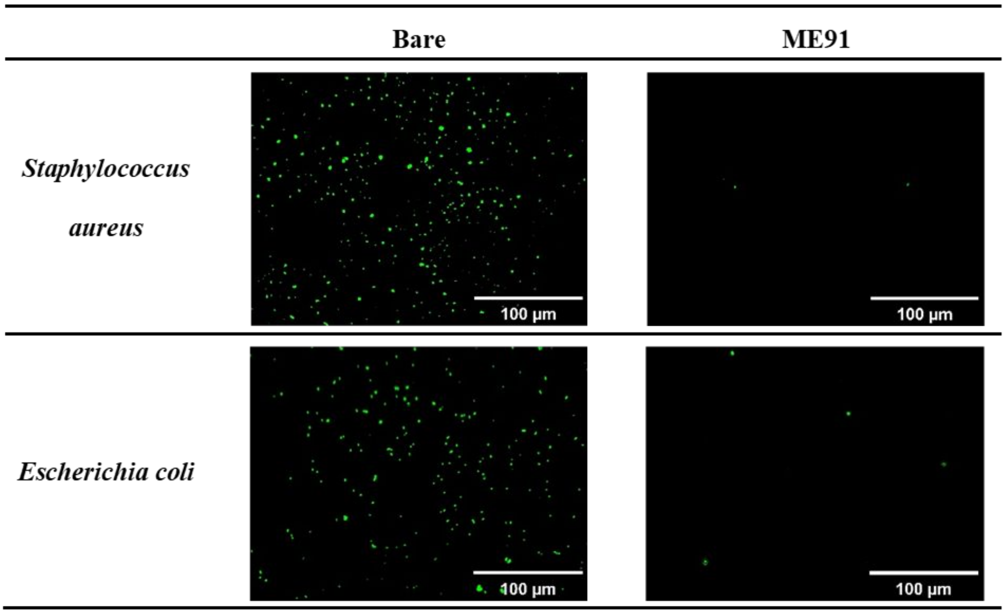

Figure S2. Representative fluorescence images for bacterial adsorption on bare and ME91-modified steel.

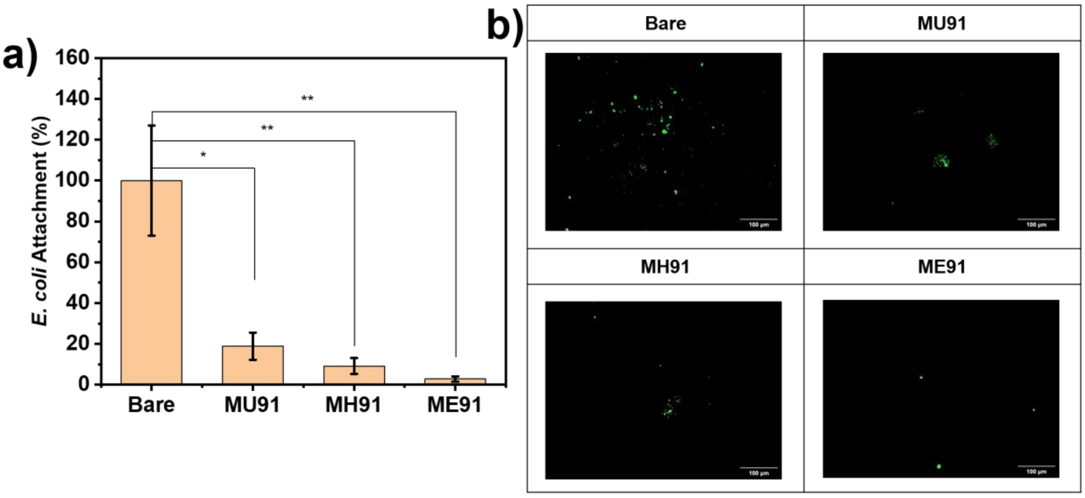

Figure S3. **Long-term *E. coli* adsorption on bare and modified stainless steel samples. (a) The attachment of bacteria was quantitatively analyzed. (b) The presence of bacteria is identified by using fluorescence microscopy. Scale bar = 100 µm. *: $p \le 0.05$; **: $p \le 0.01$**

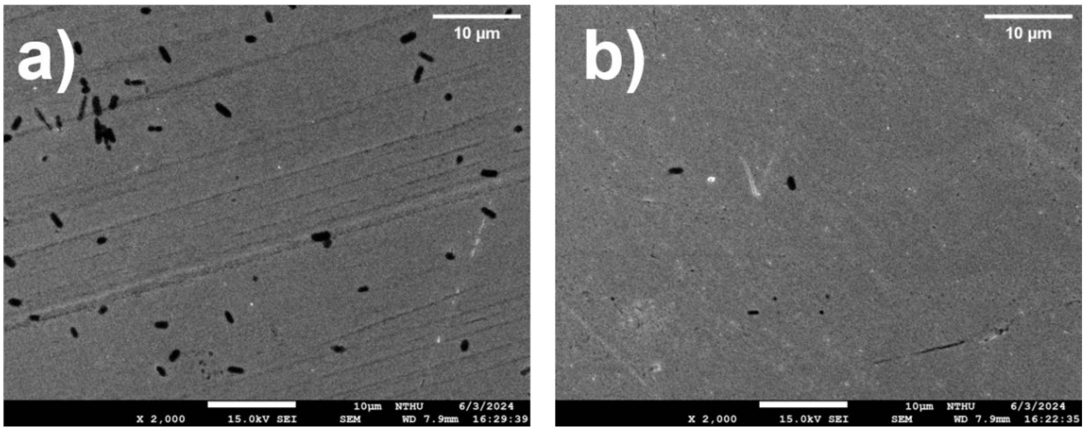

Figure S4. **SEM images of *E. coli* on bare (a) and ME91-modified (b) stainless steel samples. Scale bar = 10 µm.**
